# *E. coli* strain-dependent lipid alterations in cocultures with endothelial cells and neutrophils modeling sepsis

**DOI:** 10.3389/fphys.2022.980460

**Published:** 2022-09-20

**Authors:** Kaushalya Amunugama, Daniel P. Pike, David A. Ford

**Affiliations:** ^1^ Edward A. Doisy Department of Biochemistry and Molecular Biology, Saint Louis University School of Medicine, St. Louis, MO, United States; ^2^ Center for Cardiovascular Research, Saint Louis University School of Medicine, St. Louis, MO, United States

**Keywords:** sepsis, *E. coli*, lipids, neutrophils, endothelial cells, infection, lipidomics

## Abstract

Dysregulated lipid metabolism is common in infection and inflammation and is a part of the complex milieu underlying the pathophysiological sequelae of disease. Sepsis is a major cause of mortality and morbidity in the world and is characterized by an exaggerated host response to an infection. Metabolic changes, including alterations in lipid metabolism, likely are important in sepsis pathophysiology. Here, we designed an *in vitro* cell culture model using endothelial cells, *E. coli*, and neutrophils to mimic sepsis in a simplified cell model. Lipid alterations were studied in the presence of the pathogenic *E. coli* strain CFT073 and non-pathogenic *E. coli* strain JM109. We employed untargeted lipidomics to first identify lipid changes and then targeted lipidomics to confirm changes. Both unique and shared lipid signatures were identified in cocultures with these *E. coli* strains. In the absence of neutrophils, the CFT073 strain elicited alterations in lysophosphatidylcholine and diglyceride molecular species during coculture while both strains led to increases in phosphatidylglycerols. Lipid alterations in these cocultures changed with the addition of neutrophils. In the presence of neutrophils with *E. coli* and endothelial cells, triglyceride increases were a unique response to the CFT073 strain while phosphatidylglycerol and diglyceride increases occurred in response to both strains. Phosphatidylethanolamine also increased in neutrophils, *E. coli* and endothelial cells cocultures, and this response was greater in the presence of the CFT073 strain. We further evaluated changes in phosphatidylethanolamine in a rat model of sepsis, which showed multiple plasma phosphatidylethanolamine molecular species were elevated shortly after the induction of sepsis. Collectively, these findings demonstrate unique lipid responses by co-cultures of *E. coli* with endothelial cells which are dependent on the *E. coli* strain as well as the presence of neutrophils. Furthermore, increases in phosphatidylethanolamine levels in CFT073 urosepsis *E. coli*, endothelial cell, neutrophil cocultures were similarly observed in the plasma of septic rats.

## Introduction

Lipids play essential roles in physiological and pathological processes. They are crucial components of cell membranes and signaling mechanisms, and they serve as energy sources. Dysregulated lipid metabolism and activation of lipid-mediated signaling pathways are observed in various disease conditions, including sepsis and COVID-19 (Gijon et al., 1995; Bruegel et al., 2012; Rival et al., 2013; Ahmad et al., 2020; Amunugama et al., 2021a; Snider et al., 2021). Plasma lipidome changes during COVID-19 have been attributed to secretory phospholipase A_2_ activity (Snider et al., 2021). Sepsis is a life-threatening organ dysfunction caused by an exaggerated immune response to an infection (Rudd et al., 2020). Sepsis disease heterogeneities, including bacterial (or viral, including SARS-CoV-2) speciation of the initial infection, may lead to specific mechanisms mediating the pathophysiological sequelae of the disease and specific biomarkers.

Systemic neutrophil activation and endothelial barrier dysfunction are common in sepsis. Neutrophils are the major white blood cells that are activated in the early stages of sepsis and are vital for microbial killing. The role of neutrophils in the pathogenesis of sepsis is complex and not completely understood. Several studies show impaired neutrophil migration to the site of inflammation, declined chemotactic responses, and decreased endothelial-leukocyte interactions during sepsis (Alves-Filho et al., 2008; Kovach and Standiford, 2012; Sae-Khow et al., 2020). Alternatively, neutrophil hyperactivation and enhanced neutrophil recruitment have been shown to improve pathogen clearance and sepsis survival (Liaw et al., 2005; Craciun et al., 2010). Additionally, due to continuous inflammatory stimuli and endotoxin exposure during sepsis, the endothelium becomes activated to promote a pro-adhesive and pro-thrombotic surface (Cepinskas and Wilson, 2008). The activated endothelium destabilizes the gap junctions and tight junctions leading to loss of the endothelial barrier (Li et al., 2020). Endothelial hyperpermeability may result in interstitial edema, which subsequently increases interstitial pressure and organ damage in sepsis (Ait-Oufella et al., 2010). Pro-thrombotic endothelium triggers platelet-leukocyte aggregates blocking the microcirculation (Joffre et al., 2020).

Although the blood-endothelial interface has an important pathophysiological role in sepsis, little is known regarding lipids at this interface in the presences of bacteria. Here we designed a simplified *in vitro* cell culture-based model to investigate lipid signatures altered under conditions mimicking sepsis. The human endothelial cell line EA.hy926 (EA) was exposed to either uroseptic CFT073 or non-pathogenic JM109 *E. coli* strains in the presence and absence of neutrophils. Untargeted and targeted lipidomics were performed to identify altered lipid profiles. These studies revealed significant changes in multiple lipid classes and lipid molecular species under the different coculture conditions tested. Furthermore, coculture of endothelial cells, neutrophils and uroseptic CFT073 *E. coli* led to increased phosphatidylethanolamine levels, which were also elevated in the plasma during an early stage of rat sepsis.

## Materials and methods

### Neutrophil preparation

Neutrophils were isolated from whole blood of healthy human donors as previously described with approval by the Saint Louis University Institutional Review Board (Amunugama et al., 2021b). Briefly, whole blood was centrifuged over a density gradient. After centrifugation, the polymorphonuclear cell band was isolated and washed with Hanks’s balanced salt solution (HBSS). The red blood cells were lysed and neutrophils were washed twice with HBSS before preparing relevant neutrophil concentrations in HBSS.

### Bacteria strains

CFT073 and JM109 *E. coli* strains were used as pathogenic and non-pathogenic strains, respectively. Overnight pre-cultured bacteria were sub-cultured in Luria-Bertani (LB) broth shaking at 260 rpm at 37°C until they reached the exponential growth phase. Bacteria cell number was calculated using pre-drawn growth curves. Bacteria were washed in HBSS and appropriate concentrations were prepared in HBSS.

### Coculture sample preparation for lipidomics

Endothelial-*E. coli* cocultures were prepared as follows. Endothelial EA.hy926 (EA) cells were plated on 6 well plates and grown to 100% confluency in Dulbecco’s Modified Eagle medium with 10% FBS. Plates were rinsed with HBSS and 20 × 10^6^/ml of CFT073 *E. coli* or JM109 *E. coli* were added in HBSS and incubated 1 or 2 h at 37°C. The EA: *E. coli* cell ratio was 1:20. Control samples were prepared by culturing EA and bacteria separately and combining them at the end of incubation. Experiments were performed using 3 biological replicates each having 3 experimental replicates. Endothelial-*E. coli*-neutrophil cocultures were prepared as follows, EA.hy926 cells were plated on 6 well plates and were rinsed with HBSS prior to the addition of 20 × 10^6^/ml CFT073 or JM109 *E. coli* in HBSS. Cultures were incubated for 1 h at 37°C. Subsequently, 2 × 10^6^ human neutrophils in 0.5 ml HBSS were added into each well, keeping the ratio of neutrophils: bacteria at 1:10 in the coculture. The EA: *E. coli*: neutrophil ratio was 1:20:2. The cultures were further incubated an additional hour at 37°C. In parallel to these coculture experiments, control experiments were performed by incubating *E. coli*, neutrophils, and EA cells separately at 37°C. Following incubation, coculture samples were rapidly scraped into glass tubes and methanol was added prior to rapid freezing on dry ice. The control samples were treated under identical conditions to coculture conditions. Coculture and control samples were stored at −80°C until lipid extraction. During the lipid extraction step, the control EA, *E. coli*, and neutrophil samples were combined to generate a single control sample. To account for the neutrophil donor variability, untargeted lipidomics samples were performed using 5 biological replicates with different neutrophil donors each having 3 experimental replicates. Targeted lipidomics experiments were performed using 3 biological replicates each having 3 experimental replicates.

### Lipid extraction for untargeted and targeted lipidomics

Lipids were extracted using a modified Bligh Dyer extraction method as previously described (Bligh and Dyer, 1959). Samples were spiked with 100 µl of internal lipid standard mix that consists of 1.5 µg phosphatidylcholine (PC) (20:0/20:0, where x:y/x:y indicates x number of carbons and y number of double bonds in *sn*-1 and *sn*-2 fatty acids esterified to the glycerol backbone), 0.3 µg phosphatidylethanolamine PE (14:0/14:0), 0.2 µg sphingomyelin (d18:1/17:0, where d indicates dihydro aliphatic group in the sphingosine backbone), 0.15 µg cholesteryl ester (17:0), 0.1 µg fatty acid (17:0), 15 ng ceramide (Cer) (17:0), 0.15 µg lysophosphatidylcholine (LPC) (17:0), 0.1 µg phosphatidylserine (PS) (14:0/14:0), 0.15 µg triglyceride (TG) (17:0/17:0/17:0), 0.2 µg phosphatidylglycerol (PG) (14:0/14:0) and 0.05 µg diglyceride (DG) (20:0/20:0). Lipid extracts were dried under nitrogen and were dissolved in methanol: isopropanol (1:1) for lipidomic analysis.

### LC conditions

Reverse-phase HPLC was performed using an Accucore C18 column (2.1 mm × 150 mm) at 35°C. Mobile phase A was comprised of 60% acetonitrile, 40% water, 10 mM ammonium formate and 0.1% formic acid. Mobile phase B was comprised of 90% isopropanol, 10% acetonitrile with 2 mM ammonium formate, and 0.02% formic acid. The HPLC gradient started at 30% B, which was held for 3 min, followed by a discontinuous gradient as follows: 1) 30% B to 60% B over 4 min; 2) 60% B to 85% B over 8 min; 3) 85% B to 100% B over 9 min; and 4) 100% B continued for 3 min. The autosampler temperature was kept at 10°C. This HPLC method was employed for both untargeted and targeted parallel reaction monitoring (PRM) analyses.

### MS conditions for untargeted and targeted lipidomics

Untargeted lipidomics and targeted PRM lipidomics were performed using a Q-Exactive mass spectrometer (Thermo Scientific). For untargeted lipidomics data-dependent mass spectrometry-mass spectrometry (ddMS^2^), the top 10 most abundant peaks from a full MS1 scan were acquired in both positive and negative ion modes. Full scan MS1 was performed with chromatogram peak width set at 7 s, scan range 200–1,200 m/z, AGC target 1 × 10^6^, resolution 70,000, and maximum injection time 246 ms. For ddMS^2^ negative ion analyses, parameters were resolution 17,500, maximum injection time 54 ms; AGC target 2 × 10^5^; isolation window 1.0 m/z; normalized collision energy (NCE) 20, 30, and 40; and dynamic exclusion at 10 s. Similar parameters were used for positive ion mode ddMS^2^ analyses, except NCE was set to 20 and 40. Ions present in blank injections were excluded.

For two-cell and three-cell coculture systems, targeted lipidomics was performed by PRM using Q-Exactive MS/MS. TG, and DG were analyzed in positive ion mode while PE and PG species were detected by negative ion mode. PC species were quantified by positive ion while negative ion mode PRM was also performed to confirm the respective fatty acids. Fatty acid composition of complex lipids was determined by MS/MS of fatty acids and fatty acid loss in negative and positive ion mode, respectively. The *m/z* of parent ion and product ion fragments used for species confirmation are given in the (Supplementary Table S1). Quantification of each lipid species was performed based on the lipid standards and normalized to the EA cells (1 × 10^6^ cells in a well) that were scraped and extracted in coculture and control samples.

### MS data processing for untargeted lipidomics

MS data were analyzed using Xcalibur Qual Browser (Thermo Scientific). Untargeted LC-MS data were processed using LipidSearch 4.1 (Thermo Scientific) (Breitkopf et al., 2017; Contrepois et al., 2018). Both positive and negative MSdd.raw (data-dependent) files were used for lipid identification. Peak areas were normalized to internal lipid standards for each lipid class. Only the lipid classes that were normalized to internal standards were selected for further analysis. Each identified lipid was manually validated by investigating MS/MS fragmentation, the compatible retention time for lipid class, and acceptable peak shape. The fragmentation pattern for each lipid class was further confirmed by running a standard mix alone. The normalized, main ion data were transferred to MetaboAnalyst 5.0 for data analysis (Chong and Xia, 2020). Data scaling was set to autoscaling (mean-centered and divided by the standard deviation of each variable). The significant species were selected using the cut-off of 1.5-fold change and *p*-value < 0.05. Following volcano plot analysis, the top-ranked lipid species were selected for targeted lipidomics using PRM.

### Bacterial survival assay

Bacterial survival in coculture conditions was assessed by first adding 100 U/ml of DNAse to eliminate cell aggregates. Then, 50 µl of the sample was diluted in pH 11 water and incubated at room temperature for 5 min to lyse cells as previously described (Parker et al., 2014). Subsequently, samples were serially diluted in HBSS and plated on LB agar plates. Following overnight incubation, colony-forming units were calculated. The bacterial survival was calculated as a percentage of the starting bacteria number.

### Lactate dehydrogenase assay

The cell viability of EA cells following bacteria exposure was determined by lactate dehydrogenase (LDH) release. After incubation with bacteria, cell media was collected and the amount of LDH was measured using an LDH kinetic assay as previously described (Freyer and Harms, 2017). The assay was performed in 96 clear well plates and the absorbance was measured at 530 nm using a multimode plate reader (Enspire). The percentage of LDH release was expressed as the proportion of LDH release in cells treated with 0.1% Triton X-100.

### Rat cecal slurry studies and plasma lipid analyses

All animal experiments were conducted with the approval of the Institutional Animal Care and Use Committee at Saint Louis University. Young adult male Sprague-Dawley (Harlan–Indianapolis, IN, United States) weighing between 270–330 g were maintained in a temperature and humidity-controlled room with a 12 h light/dark cycle and unrestricted access to chow and water. Cecal slurry (CS) was prepared from donor male Sprague-Dawley rats (Pike et al., 2020). Rats were ip administered either CS (15 ml/kg) or 15% glycerol vehicle control in a total volume of 20 ml/kg, with the remaining 5 ml/kg being sterile saline (B Braun Medical, Bethlehem, PA, United States). At the time of CS administration, animals were administered a concurrent 30 ml/kg dose of subcutaneous sterile saline. Four and 8 h following CS injection, rats were euthanized, and blood was collected *via* cardiac puncture. An additional cohort of CS septic rats were treated 8 h following CS injection with both 25 mg/kg ceftriaxone (Hospira) in sterile saline (im) and a subcutaneous 30 ml/kg dose of sterile saline. In this cohort at 12 h following CS injection, rats were euthanized, and blood was collected *via* cardiac puncture. This model of sepsis has previously been shown to have an approximately 25% survival rate following 3 days (Pike et al., 2020). Without ceftriaxone and supplemental fluids at 8 h, less than 10% survival is observed following 20 h of CS injection. Plasma was immediately prepared and then stored at −80°C within 45 min of blood collection. To minimize freeze thaw cycles to two times or less, plasma was stored in aliquots. Rats were euthanized by injecting 0.5 ml Somnasol (390 mg/ml sodium pentobarbital and 50 mg/ml phenytoin sodium), ip followed by thoracotomy.

Plasma lipids were extracted in the presence of internal standards as described for coculture analyses. PC and PE molecular species were detected by selected reaction monitoring with an Altis TSQ mass spectrometer equipped with a Vanquish UHPLC System (Thermo Scientific). Lipids were separated on an Accucore C30 column 2.1 mm × 150 mm (Thermo Scientific) with mobile phase A comprised of 60% acetonitrile, 40% water, 10 mM ammonium formate, and 0.1% formic acid and mobile phase B comprised of 90% isopropanol, 10% acetonitrile with 2 mM ammonium formate, and 0.02% formic acid. Initial conditions were 30% B with a discontinuous gradient to 100% B at a flow rate of 0.260 ml/min.

### Data analysis and statistics

Student’s *t*-test was used to compare two groups while one-way analysis of variance with Tukey’s post hoc analysis and Dunnett’s post hoc test were used to compare three or more multiple comparisons. All data were represented as mean with standard deviation with averages of 3 biological replicates unless otherwise indicated. Following untargeted data processing by LipidSearch 4.1, data was transferred to MetaboAnalyst 5.0 to perform additional statistical analyses. GraphPad Prism was used for all other statistical analyses.

## Results

### Untargeted lipidomics reveal both unique and shared lipidomic signatures following endothelial exposure to CFT073 and JM109 *E. coli*

**FIGURE 1:**
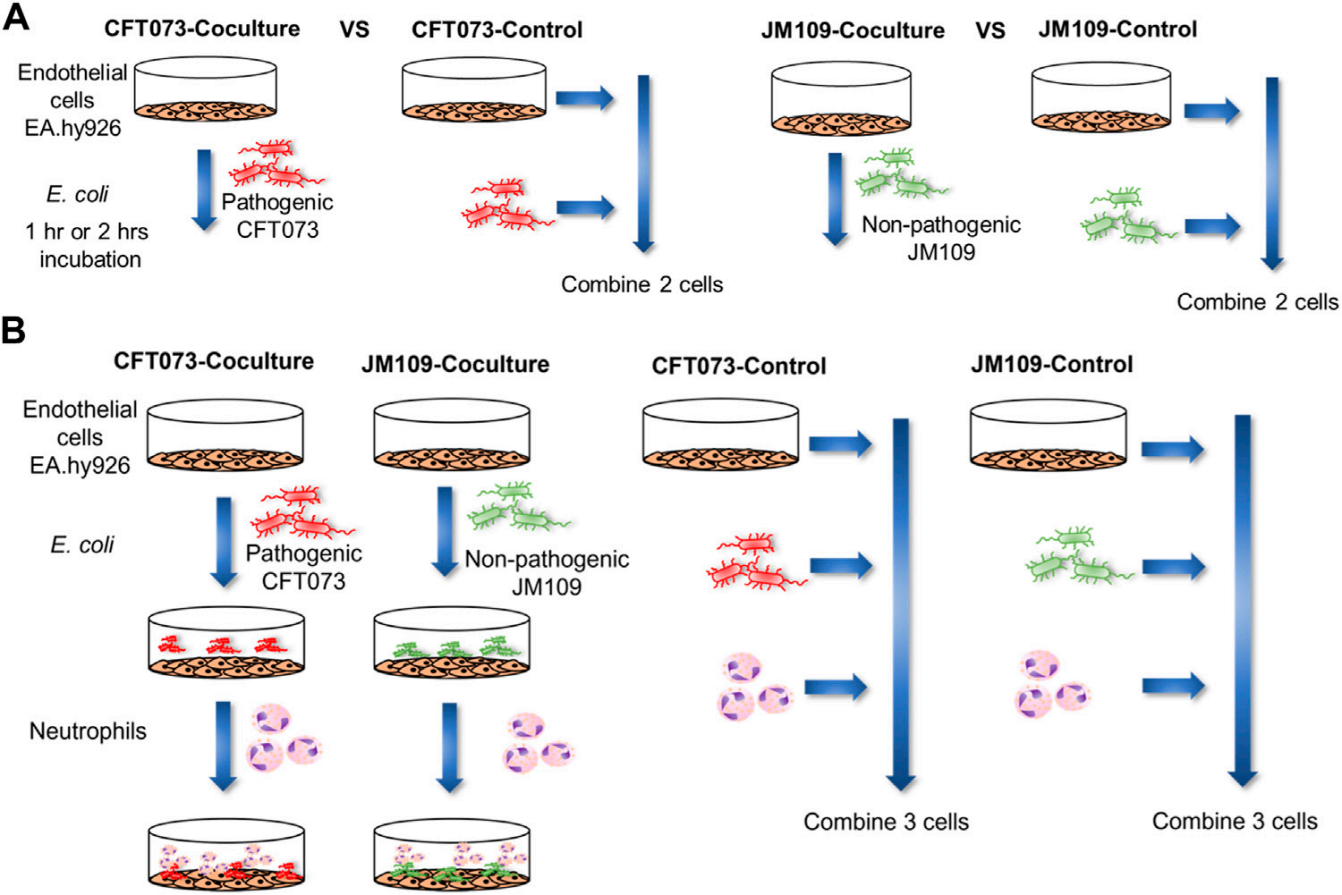
Schematic illustration of the workflow for coculture lipidomics. **(A)** 2 cell system: EA hy.926 cells (EA) were exposed to 20 × 10^6^ of either CFT073 or JM109 *E. coli* for 1 or 2 h at 37°C. The cell ratio of EA:*E. coli* was 1:20. Control samples for this system were prepared by combining *E. coli*, and EA cells that were incubated separately. **(B)** 3-cell system: EA cells were exposed to 20 × 10^6^ of either CFT073 or JM109 *E. coli* for 1 h at 37°C. Subsequently, 2 × 10^6^ neutrophils were added and further incubated for 1 h at 37°C. The cell number ratio of EA:*E. coli*:neutrophils was 1:20:2. Control samples were prepared by combining *E. coli*, neutrophils and EA cells that were incubated separately. At the end of incubation, cells and media were collected for lipidomic studies.

**FIGURE 2:**
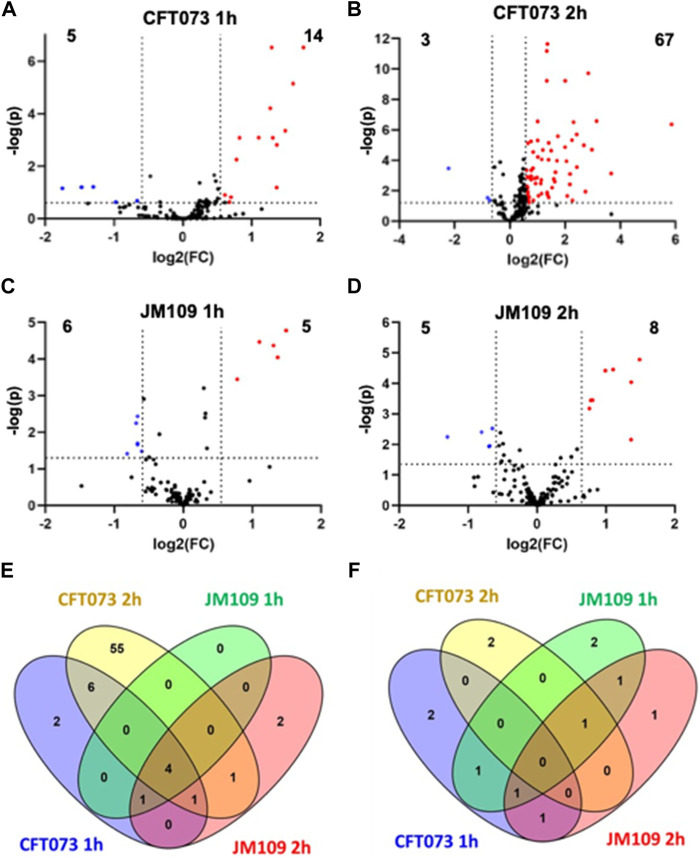
Untargeted lipidomics reveal changes in lipidomes during bacteria endothelial coculture (2-cell system). Volcano plots are shown comparing coculture conditions to the respective combined control cultures. CFT073 cocultures were compared to CFT073 combined control at 1 h **(A)** and 2 h **(B)** coculture intervals. JM109 cocultures were compared to JM109 combined control at 1 h **(C)** and 2 h **(D)** coculture intervals. Statistical significance for A, B, C and D were evaluated by *t*-test (*p-*value < 0.05) and fold change (FC) threshold at 1.5 **(E)** Venn diagram representing increased lipid molecular species in CFT073 coculture and JM109 coculture at 1 and 2 h. **(F)** Venn diagram representing decreased lipid molecular species in CFT073 coculture and JM109 coculture at 1 and 2 h.

Initial studies examined changes in the endothelial lipidome in response to bacteria exposure with cocultures of either CFT073 or JM109 *E. coli* strains with EA.hy926 cells as illustrated in Figure 1A. Untargeted lipidomics was performed to study the array of lipidome changes and was followed by targeted lipidomics to confirm the identities and quantify lipids. Untargeted lipidomics analyses revealed the CFT073 *E. coli* strain induces more lipid changes compared to the JM109 strain following both 1 and 2 h exposures (Figures 2A–D). Exposure to CFT073 for 1 h resulted in increased levels of 14 lipid molecular species (Figure 2A) while 2 h exposures led to increased levels of 67 lipid molecular species (Figure 2B). In contrast, at both 1 and 2 h exposures the JM109 strain led to increases in 5 and 8 lipid molecular species, respectively (Figures 2C,D).

To characterize lipids that differ between CFT073 and JM109 exposure, the significantly increased and decreased lipids were compared between the two *E. coli* strains (Figures 2E,F; Supplementary Tables S2, S3). There were four lipids increased in all coculture conditions (common intersection in Figure 2E), which were all PG molecular species including PG 16:0/16:1, PG 16:0/18:1, PG 16:1/18:1, and PG 18:1/18:1. Fifty-five unique lipid species were identified in the CFT073 2 h coculture condition. These lipids included TG (6 species), DG (16 species), Cer (15 species), PE (11 species), and PC (6 species). However, there were few common lipid molecular species that decreased under these four coculture conditions. Several DG and PE molecular species were decreased in CFT073 strain and JM109 strain exposures to EA cells (Figure 2F).

**FIGURE 3:**
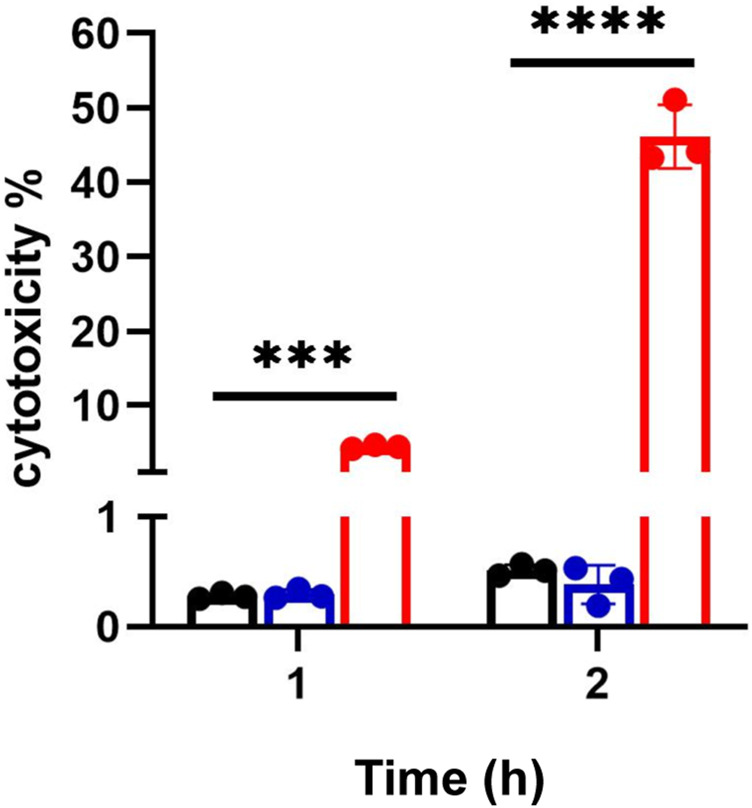
Bacteria mediated endothelial cell death. EA cells were exposed to either CFT073 (red) or JM109 (blue) at EA:*E. coli* cell ratio of 1:20 for indicated times at 37°C. At the end of incubation, media were collected and LDH assay was performed as described in “*Materials and Methods*”. Statistics were performed using one way ANOVA, *n* = 3, *p* value: ****p < 0.0001, ***p < 0.001.

Cer increases following 2 h coculture suggested cell death under these conditions. Accordingly, we investigated cell death under these conditions. Figure 3 shows CFT073 causes ∼9 % and ∼45% cytotoxicity at 1 and 2 h, respectively. JM109 did not cause cell death (below 1% cytotoxicity) at either incubation time (Figure 3). Accordingly, we selected 1 h coculture conditions for further targeted lipidomic analysis of lipid classes identified by untargeted analysis to detect lipid changes prior to cell death.

**FIGURE 4:**
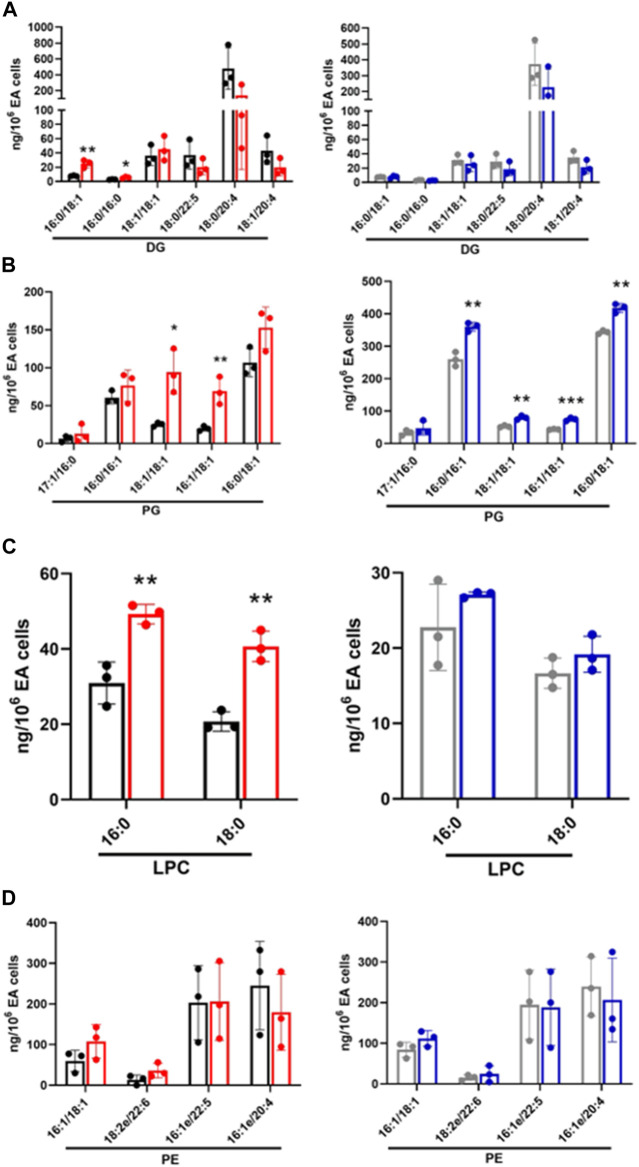
Targeted lipidomics of endothelial and *E. coli* coculture following a 1 h incubation. Following untargeted lipidomic analysis, significantly altered lipids detected between cocultures and control conditions following a 1 h incubation were selected for targeted lipidomics. Lipid species were analyzed by PRM using Q Exactive MS/MS. DG **(A)**, PG **(B)**, and LPC **(C)**, and PE **(D)** were measured in CFT073 coculture (red), CFT073 combined control (black), JM109 coculture (blue) and JM109 combined control samples (gray). Statistics were performed using unpaired *t*-test, *n* = 3 for average of 3 biological replicates, *p* value: ***p < 0.001, **p < 0.01, *p < 0.05.

Increased or decreased lipids identified in untargeted analysis were subjected to targeted lipidomics using PRM. Targeted analysis confirmed DG 16:0/18:1 and DG 16:0/16:0 levels have a significant increase in CFT073 coculture while several other DG species trended toward decreasing (Figure 4A). DG species in this targeted analysis were unchanged in JM109 cocultures. Most of the PG species were significantly increased in JM109 cocultures compared to the control condition (Figure 4B). Overall, PG levels were lower in CFT073 cocultures compared to JM109 cell cocultures. However, the magnitude of increases in 16:1/18:1 and 18:1/18:1 PG levels in response to coculture conditions with CFT073 in respect to control culture conditions was much greater compared to that in JM109. Additionally, LPC 16:0 and LPC 18:0 levels were elevated only in CFT073 coculture (Figure 4C). Interestingly although significant changes in PE were suggested by untargeted lipidomics, changes in PE species were not confirmed during both of the coculture conditions using targeted analyses (Figure 4D).

### Bacteria species-dependent lipidomic alterations in the presence of neutrophils

**FIGURE 5:**
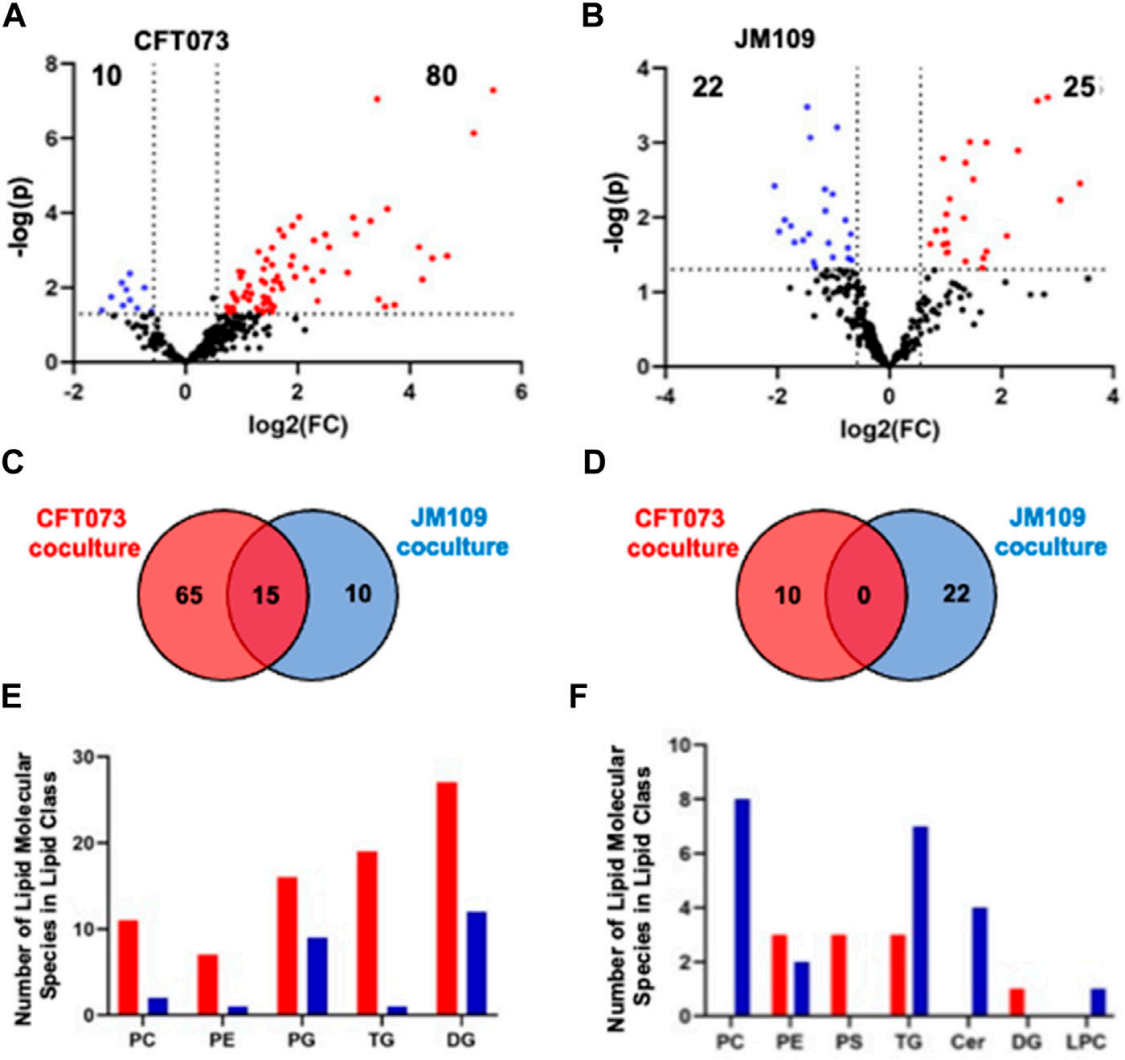
Untargeted lipidomics reveal altered lipid levels during bacteria coculture with endothelial cells and neutrophils. **(A)** Volcano plot comparing CFT073 coculture with CFT073 combined control. **(B)** Volcano plot of JM109 coculture compared to JM109 combined control. Statistical significance was evaluated by *t*-test (*p-*value < 0.05) and fold change (FC) threshold at 1.5. The number of increased and decreased lipids are shown in their respective quadrants. **(C)** Venn diagram representing increased lipid molecular species for comparisons between CFT073 coculture and JM109 coculture. **(D)** Decreased lipid species for comparisons between CFT073 and JM109 cocultures. **(E)** Increased lipid molecular species by lipid class from either CFT073 (red) and JM109 (blue) cocultures. **(F)** Decreased lipid molecular species by lipid class from either CFT073 or JM109 cocultures.

To characterize the coculture lipidome in the presence of neutrophils following initial bacteria coculture with endothelial cells we designed a 3-cell system as illustrated in Figure 1B. As in our studies with EA cells and *E. coli,* we first analyzed changes in the lipidome using untargeted approaches followed by targeted methods. The untargeted lipidomic analysis identified a total of 270 annotated lipid species across 11 lipid classes in both CFT073 and JM109 coculture conditions. Eighty lipid species were significantly elevated in CFT073 coculture compared to the CFT073 combined control (Figure 5A). In contrast, JM109 coculture condition resulted in only 25 significantly elevated lipid species (Figure 5B). However, JM coculture showed a higher number of decreased lipid species (22 lipids) while CFT073 coculture showed only 10 decreased lipid species (Figures 5A,B).

To determine unique and shared lipid signatures due to bacteria strain-specific cocultures, we then compared increased lipid species in CFT073 and JM109 cocultures (Figure 5C). Fifteen lipid species were commonly increased in CFT073 and JM109 cocultures. These common increased lipid species exclusively consist of DG and PG species (Supplementary Table S4). In contrast, there were no common decreased lipid species in comparisons between CFT073 and JM109 coculture conditions (Figure 5D; Supplementary Table S5). We then classified significantly altered lipids into lipid classes. Lipid species that were elevated in CFT073 coculture were distributed among TG, DG, PG, PC, and PE (Figure 5E). Most of the elevated lipids belong to TG and DG classes. The decreased lipids included additional lipid classes such as LPC, PS, and Cer. Interestingly, PC, Cer, and LPC were decreased in JM109 coculture while PS was decreased in CFT073 coculture (Figure 5F).

**FIGURE 6:**
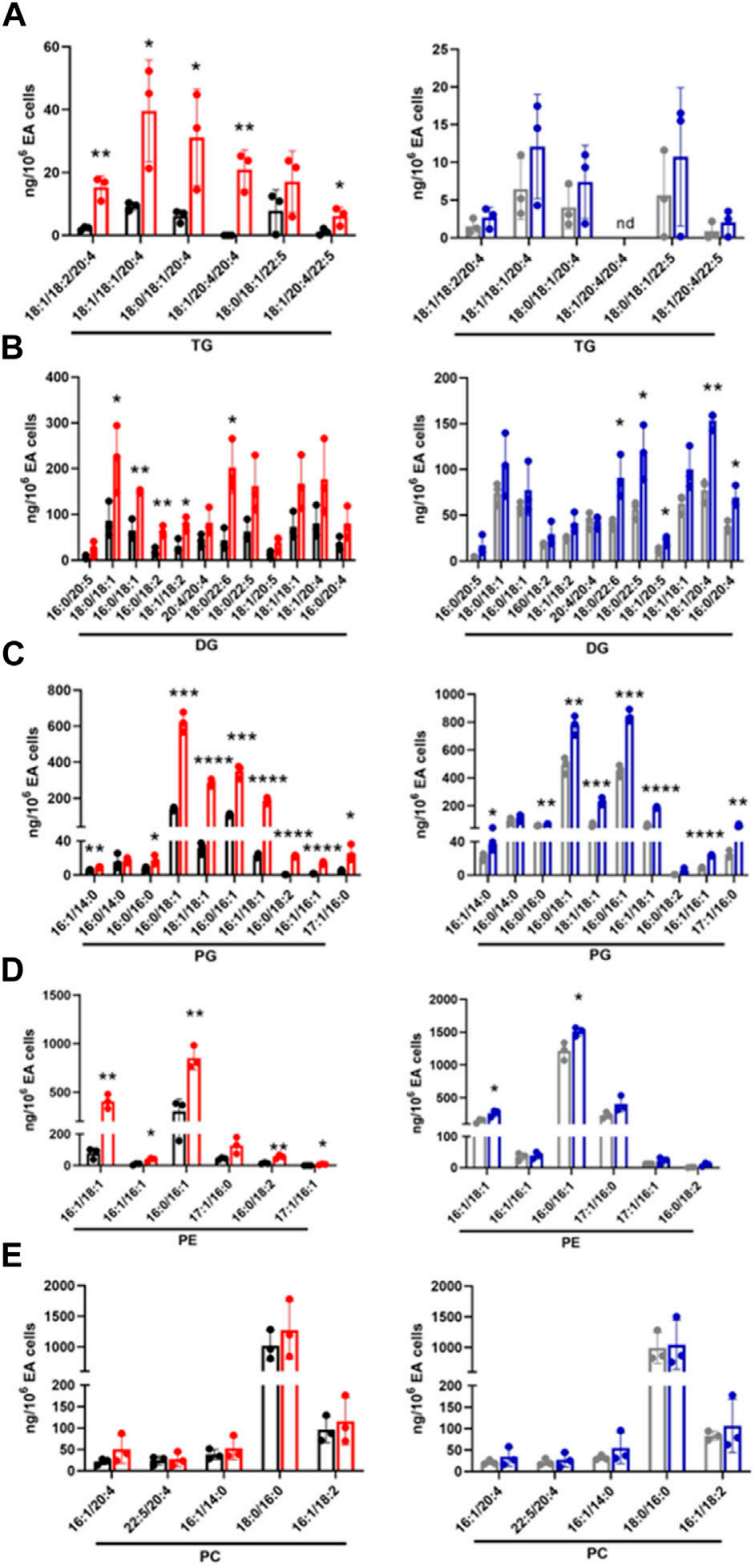
Targeted analyses based on targets identified in unbiased analyses of endothelial, *E. coli* and neutrophil coculture (3-cell system). Lipid molecular species identified following untargeted lipidomics of endothelial, *E. coli* and neutrophil cocultures (3-cell system) were quantified using PRM. TG **(A)**, DG **(B)**, PG **(C)**, PE **(D)**, and PC **(E)** were measured in CFT073 coculture (red), CFT073 combined control (black), JM109 coculture (blue) and JM109 combined control samples (gray). Statistics were performed using unpaired *t*-test, *n* = 3 for average of 3 biological replicates, *p* value: ****p < 0.0001, ***p < 0.001, **p < 0.01, *p < 0.05.

**FIGURE 7:**
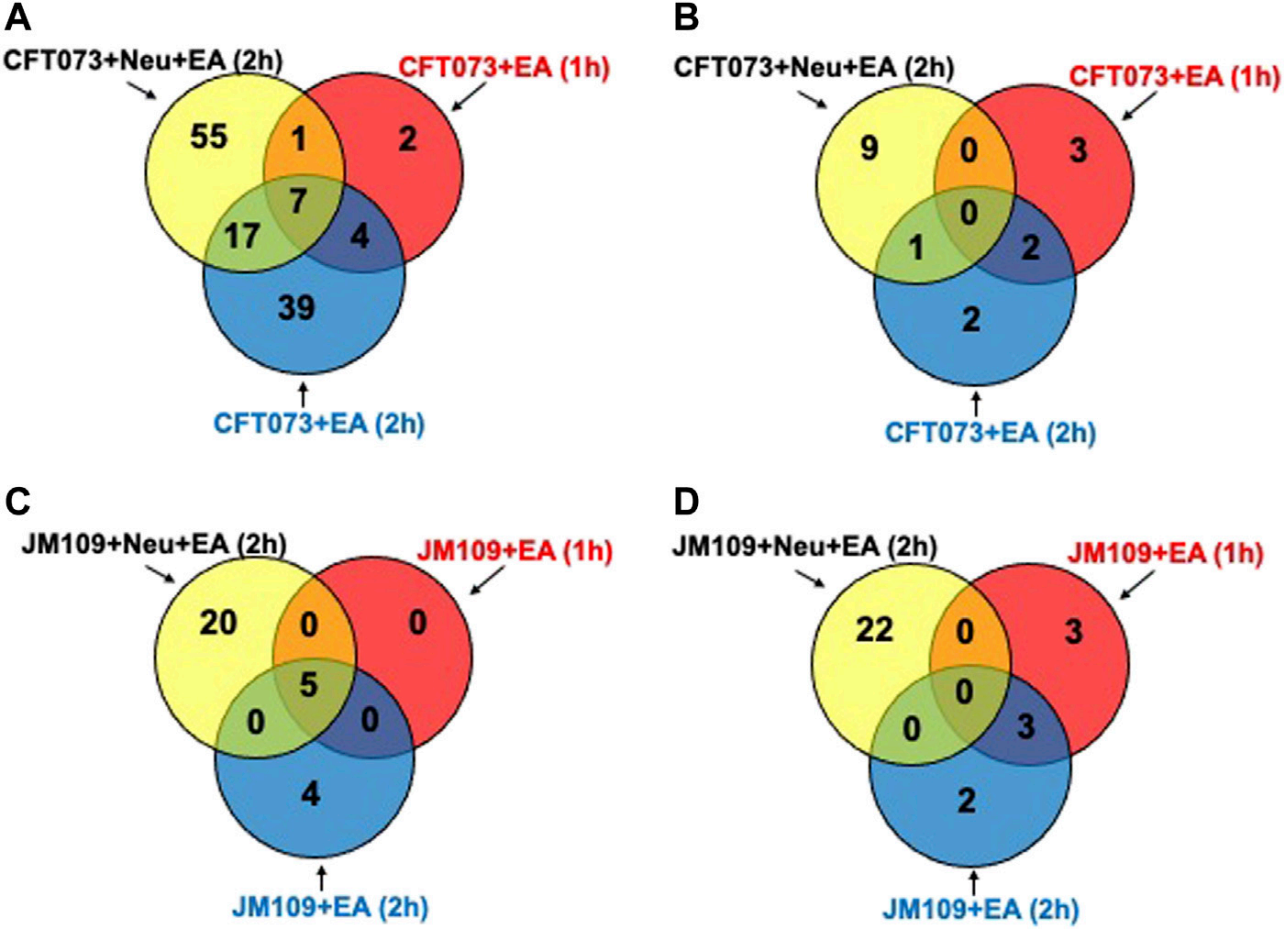
Differences in bacterial strain response with and without neutrophils at early and late time. Venn diagram representing increased **(A)** or decreased **(B)** lipid molecular species for comparisons between CFT073 cultured with EA cells with or without neutrophils at early and late time. Venn diagram representing increased **(C)** or decreased **(D)** lipid molecular species for comparisons between JM109 cultured with EA cells with or without neutrophils at early and late time.

Next, targeted lipidomics using PRM was performed to confirm and quantify top-ranked lipids identified in untargeted lipidomics from the 3-cell systems. Except for TG 18:0/18:1/22:5, TG targets containing polyunsaturated fatty acids were significantly increased in the CFT073 coculture system (Figure 6A). TG species were elevated approximately 5-fold compared to controls and were 4-fold higher than that of the JM109 coculture system. Consistent with untargeted analysis, none of the TG species were significantly increased in JM109 coculture. In general, multiple DG species were increased in both CFT073 and JM109 cocultures compared to their controls (Figure 6B). DG 18:0/22:6 was increased in both CFT073 and JM109 cocultures while some DG species were uniquely increased in either the CFT073 or JM109 coculture condition. Overall, CFT073 cocultures showed a higher levels of DG species compared to JM109 cocultures. In cocultures with either CFT073 or JM109, PGs were significantly increased (Figure 6C) compared to controls, which confirmed untargeted lipidomic analyses. Among ten PG species tested, PG 16:0/18:1, PG18:1/18:1, PG 16:0/16:1, and PG 16:1/18:1 showed an approximately 2-fold increase in both CFT073 and JM109 cocultures compared to controls. Targeted lipidomic analysis of CFT073 cocultures demonstrated significant increases in all PE species tested except PE 17:1/16:0 (Figure 6D). PE 16:1/18:1 and PE 16:0/16:1 were significantly elevated in JM109 cocultures. Although untargeted analyses indicated several PC species were increased in CFT073 and JM109 cocultures, targeted analyses did not confirm changes in levels of these PC species (Figure 6E). Additionally, the differences in strain response with and without neutrophils were assessed at the 1 and 2 h time points (Figures 7A–D; Supplementary Tables S6–S9). These data highlight that more lipid species were increased or decreased when neutrophils were added to either CFT073 or JM109 cultured with EA cells.

### Bacterial survival in coculture conditions

**FIGURE 8:**
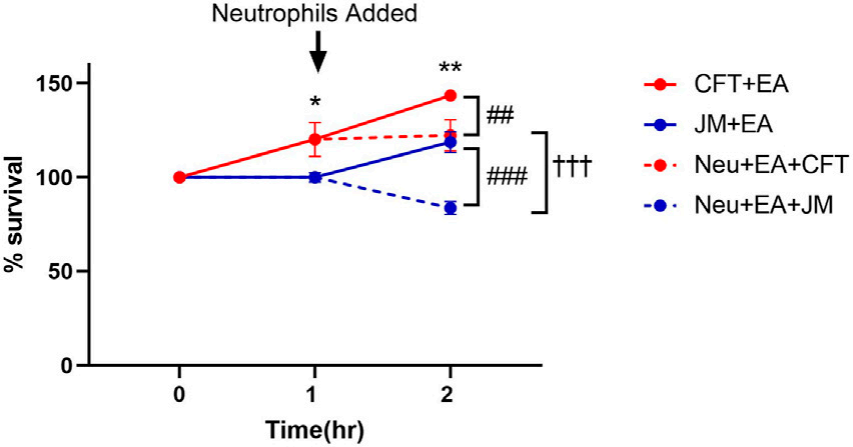
Bacterial survival during coculture conditions. Growth of CFT073 and JM109 bacteria in coculture conditions were analyzed by plating on LB agar plates as described in “*Materials and Methods*”. Bacteria survival % was calculated compared to the starting bacteria in the cultures. Data represent average of 3 biological replicates. Statistics were performed using unpaired *t*-test and one way ANOVA. *p* value: **p < 0.01, *p < 0.05 indicate CFT073 cocultured with endothelial cells (EA) compared to JM109 cocultured with EA cells. *p* value: ^###^ *p* < 0.001 ^##^ *p* < 0.01 indicate CFT073 or JM109 cocultured with EA cells compared to neutrophils added in the cocultures. *p* value: ^†††^ *p* < 0.001 indicate coculture of CFT073, EA cells with neutrophils compared to coculture of JM109, EA cells with neutrophils.

To explore the potential cause of the disparity of lipidomic profiles in these coculture systems including neutrophils, bacterial survival was assessed. Previous studies have shown that CFT073 *E. coli* is capable of adhesion, invasion, and colonization in epithelial cells and resistance to neutrophil killing mechanisms (Li et al., 2019) (Amunugama et al., 2021b). Thus, we tested the hypothesis that CFT073 *E. coli* survival is greater in the presence of neutrophils compared to that of JM109 *E. coli*. Figure 8 shows CFT073 *E. coli* survived in the presence of EA cells and neutrophils. In contrast, JM109 *E. coli* did not proliferate in the presence of EA cells and had reduced survival with the addition of neutrophils.

### Plasma phosphatidylethanolamine and phosphatidylcholine levels in septic rats

**FIGURE 9:**
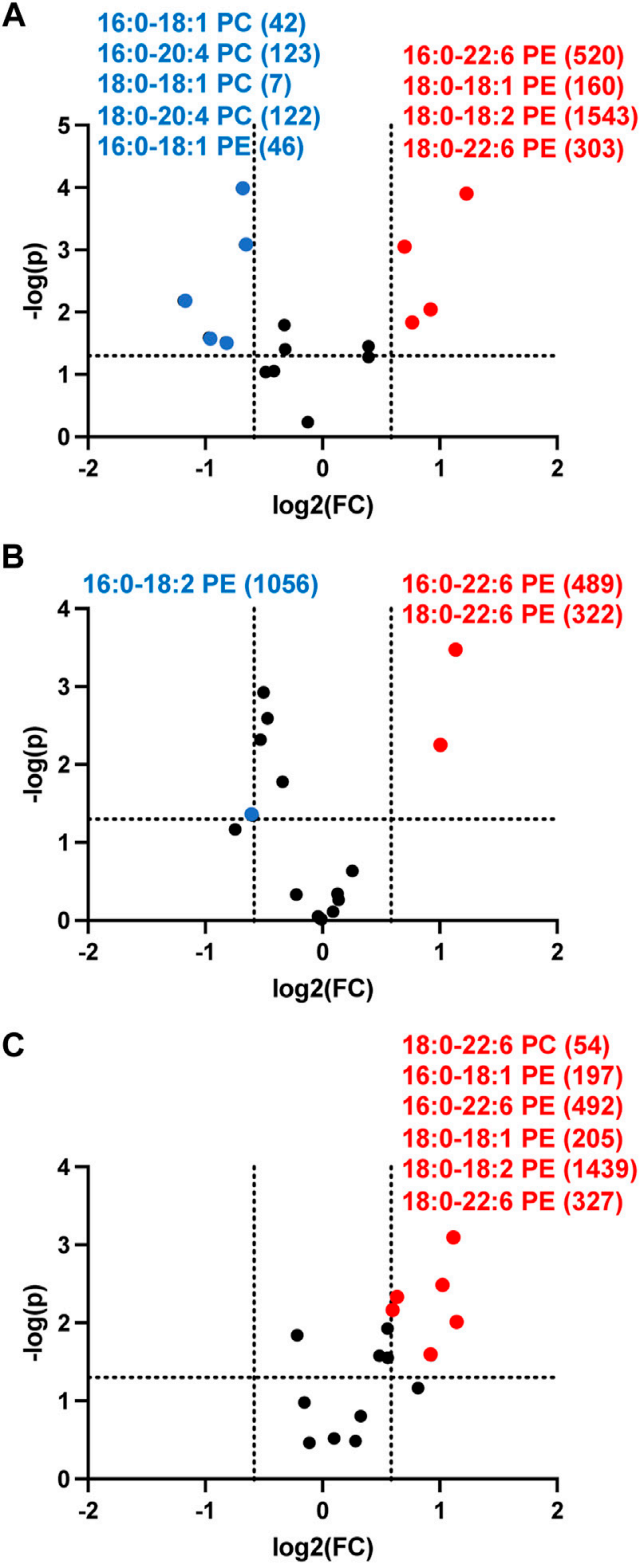
Volcano plot showing disparate changes in plasma phosphatidylethanolamine and phosphatidylcholine in septic rats. Rats were injected with cecal slurry (*n* = 5) to elicit sepsis or vehicle (*n* = 6) as described in “*Materials and Methods*”. Plasma was collected 4 h **(A)**, 8 h **(B)**, and 12 h **(C)** following cecal slurry treatment, and lipids were extracted and subjected to targeted analyses for PE and PC levels. Statistical significance was evaluated by *t*-test (*p-*value < 0.05) and fold change (FC) threshold at 1.5. (x following indicated molecular species) indicates mean value for cecal slurry treatment rat plasma levels in either nM or μM for indicated PE or PC molecular species, respectively. Blue and red data points represent molecular species that significantly decrease and increase, respectively and meet the FC criteria of 1.5.

Since targeted analyses showed PE levels were increased while PC levels did not change in the 3-cell coculture system we examined changes in these lipids in the more complex *in vivo* setting of rat sepsis. Four hours following sepsis induction by ip injection of cecal slurry, multiple rat plasma PE molecular species levels were increased compared to vehicle treated rats (Figure 9A). In contrast multiple PC molecular species levels decreased in septic animals (Figure 9A). In our analyses we were unable to detect appreciable amounts of PG in the plasma, and thus we were unable to followup on this target which was observed to change in the 3-cell coculture studies. We further assessed changes in PE and PC at 8 and 12 h following cecal slurry injection. The trend of an increase in plasma PE observed at 4 h continued at 8 and 12 h post cecal slurry injection (Figures 9B,C). One difference over time was that plasma PC levels were no longer elevated 12 h post cecal slurry injection.

## Discussion

Understanding changes in lipid levels elicited by the complex interaction of neutrophils with endothelium in the presence of bacteria have the potential to provide new insights into complex metabolic changes during bacterial challenge as well as potentially lead to the discovery of lipid biomarkers for sepsis and infection. The use of 2-cell and 3-cell coculture systems allowed us to identify lipid changes caused by bacteria interaction with host cells in the presence and absence of neutrophils. Our cell model was designed considering *in vivo* infection events, where bacteria first interact with endothelial cells and later neutrophils reach the site of inflammation for the clearance of bacteria. Unique and shared lipidomic signatures were observed during CFT073 and JM109 exposure to EA cells in the presence and absence of neutrophils. However, the CFT073 urosepsis *E. coli* strain caused more lipid changes compared to JM109 non-pathogenic *E. coli* in the presence of both EA cells and neutrophils. Most of the increased lipids are in the TG, DG, PG, and PE lipid classes. Further studies in an *in vivo* rat sepsis model also showed increases in PE levels in the plasma 4–12 h after the induction of sepsis.

Circulating lipid levels are dysregulated during infections including sepsis (Kitchens et al., 2003; Khovidhunkit et al., 2004; Tam, 2013; Amunugama et al., 2021a). For instance, Lee et al. (2015) showed sepsis-non survivors had decreased serum TG concentrations compared to sepsis survivors, and the TG levels were associated with sepsis mortality. Early studies have shown that gram-negative bacteria resulted in marked increases in serum TG concentrations (Gallin et al., 1969). Similarly, the 3-cell coculture system with the uroseptic CFT073 *E. coli* strain led to increased TG levels. These changes in TG levels were not observed in the 3-cell coculture system with the non-pathogenic JM109 *E. coli* strain. Several studies demonstrated that elevated TG level is linked with endothelial dysfunction, oxidative stress, apoptosis, and inflammation (Eiselein et al., 2007; Kajikawa and Higashi, 2019; Gorzelak-Pabiś et al., 2020). Moreover, TG-rich lipoproteins induce neutrophil activation (Alipour et al., 2008). Several DGs were significantly elevated in both CFT073 and JM109 cocultures. DGs increased with CFT073 incubated with EA cells in the absence of neutrophils. DG levels increased to much greater levels when neutrophils were added to either CFT073 or JM109 cocultures with EA cells.

Significant changes in phospholipids were revealed under coculture conditions, including PG and PE lipid classes. Importantly, we found plasma levels of multiple PE molecular species to be elevated in early stages of rat sepsis. This may be an important new finding since there is limited information on the role of PE in inflammation. For example, one report indicates PE facilitates the binding of *E. coli* to the cell membranes and induces apoptosis (Barnett Foster et al., 2000). Further studies need to be conducted to understand the role of PE in bacteria-endothelial-neutrophil interactions. Using our lipid analytical platform, we did not detect appreciable levels of plasma PG, which make it difficult to accurately determine whether plasma PG levels are altered in our rat sepsis model.

**FIGURE 10:**
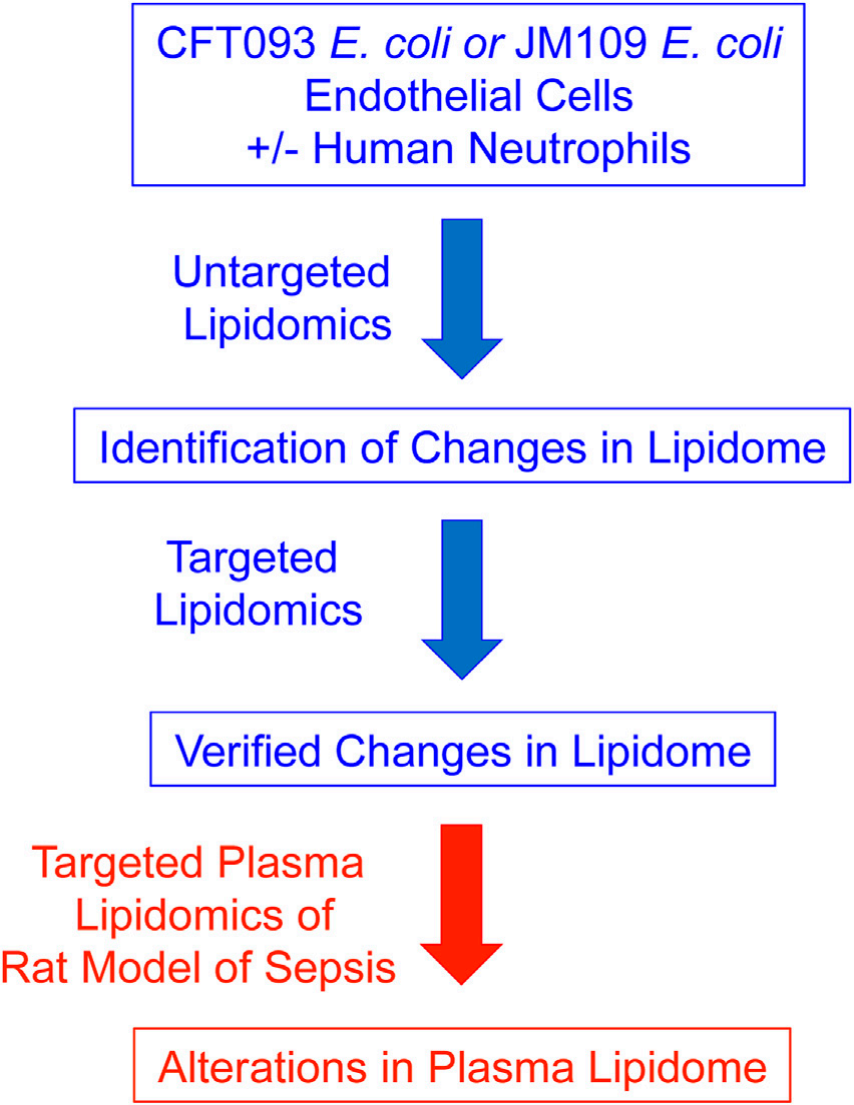
Schematic of Strategy for the Identification of Lipidomic Changes during Sepsis. The cell culture models depicted in Figure 1 were used to determine lipidomic changes first by untargeted lipidomics and subsequent verification of lipid changes by targeted lipidomics. The PC and PE molecular species targets were then examined in the plasma of septic rats.

Our strategy in these studies was to first perform untargeted lipidomics in a cell model of sepsis followed by confirmation by targeted analyses and subsequent evaluation of targets in a rat model of sepsis (Figure 10). While most candidate lipids detected using untargeted approaches were confirmed by targeted approaches, the increases in PC levels detected by untargeted lipidomics could not be confirmed by targeted lipidomics. MS-based targeted or untargeted lipidomics approaches have their distinct advantages and limitations (Cajka and Fiehn, 2016). We took advantage of untargeted lipidomics to efficiently and robustly identifying a large array of lipid species, then used targeted lipidomics to confirm and quantify candidate lipids (Telenga et al., 2014; Contrepois et al., 2018). Studies have shown that untargeted lipidomics approaches may have potential false positive and negative predictions caused by data processing tools and in-source fragmentation (Xu et al., 2018). Overall, these findings demonstrate the advantage of performing untargeted lipidomics to identify candidate targets but highlight the need to confirm lipid species by subsequent targeted approaches.

There are hundreds to thousands of lipid species that can be altered depending on physiological, pathophysiological and environmental conditions. Identifying these lipids is a daunting task using biased approaches. The evolution of high-resolution mass spectrometry and the development of lipid cloud mass spectrometry libraries has led to the ability to perform untargeted lipidomics to discover changes in lipid composition under different parameters and perhaps discover lipids that are important in biological processes or serve as biomarkers. As shown in this study confirming these candidates by subsequent targeted lipidomics approaches is important to verify these candidates. We applied these principles to find unique lipid species, which accumulate in cocultures of endothelial cells with two different strains of *E. coli* leading to the production of unique lipids. Pathogenic CFT073 *E. coli*, compared to non-pathogenic JM109 *E. coli*, led to the most profound changes in lipids. Neutrophils enhanced the increased production of lipid species in cocultures. The *E. coli*, endothelial cell, neutrophil coculture system was designed to model changes that could occur during sepsis. The role of lipid species identified in cocultures need to be further examined as mediators or protectants of endothelial cell death and neutrophil-mediated bacterial killing. While we recognize this coculture system lacks the complexity of *in vivo* responses during sepsis, many of the lipid changes observed in the coculture model have been observed in plasma during sepsis by others as discussed above. Furthermore, the observation of increased PE levels in CFT073 *E. coli*, neutrophil, endothelial cell cocultures led to our finding that plasma PE levels, but not PC levels, were elevated 4 h after the induction of rat sepsis. The pathophysiological significance of the increase of PE during sepsis needs to be further examined. Furthermore, the mechanisms leading to increased plasma PE levels in contrast to decreased plasma PC levels need to be investigated. Finally, the possibility that plasma PE levels are associated with sepsis outcomes needs to be examined in future studies.

## Data Availability

The raw data supporting the conclusion of this article will be made available by the authors, without undue reservation.
