# Supplementary material for: E. coli strain-dependent lipid alterations in cocultures with endothelial cells and neutrophils modeling sepsis
Source: Front Physiol. 2022 Sep 20;13:980460. doi: 10.3389/fphys.2022.980460 (PMC9530349; doi:10.3389/fphys.2022.980460)
Supplement: Supplementary file 1 [file Table1.DOCX]

**Table S1: Parent ion and product ion *m/z* used in PRM.** Phosphatidylethanolamine (PE), Triglycerides (TG), diglycerides (DG), phosphatidylglycerol (PG), phosphatidylcholine (PC), phosphatidylcholine (LPC)

| **Lipid Species** | ***m/z* of the adduct** | **Collision energy** | **Ion Fragments used for confirmation** | **Product ion used for quantification** |
| --- | --- | --- | --- | --- |
| **PG** | **[M-H]^-^** |  |  | **[phosphoglycerol-H2O]−** |
| PG (14:0/14:0) standard | 665.4407 | 30 | 153, 227 | 153 |
| PG (16:1/14:0) | 691.4556 | 30 | 153,227,253 | 153 |
| PG (16:0/14:0) | 693.4715 | 30 | 153,227,255 | 153 |
| PG (16:0/16:0) | 721.503 | 30 | 153,255 | 153 |
| PG (16:0/18:1) | 747.5179 | 30 | 153,255,281 | 153 |
| PG (18:1/18:1) | 773.5338 | 30 | 153,281 | 153 |
| PG (16:0/16:1) | 719.4875 | 30 | 153,253,255 | 153 |
| PG (16:1/18:1) | 745.5029 | 30 | 153,253,281 | 153 |
| PG (16:0/18:2) | 745.502 | 30 | 153,255,279 | 153 |
| PG (16:1/16:1) | 717.4715 | 30 | 153,253 | 153 |
| PG (17:1/16:0) | 733.5029 | 30 | 153,255,267 | 153 |
|  | | | | |
| **TG** | **[M+NH_4_] ^+^** |  |  | **NL(FA) -H+NH_4_^+^** |
| TG (17:1/17:1/17:1) standard | 860.7702 | 25 | 575,251,325 | 575 |
| TG (18:1/18:2/20:4) | 922.7857 | 25 | 601,339,337,623,625 | 601 |
| TG (18:1/18:1/20:4) | 924.8018 | 25 | 603,625,339,287,265 | 603 |
| TG (18:0/18:1/20:4) | 926.8171 | 25 | 605,625,627,287 | 605 |
| TG (18:1/20:4/20:4) | 946.7857 | 25 | 625,647,287,339,287 | 625 |
| TG (18:0/18:1/22:5) | 952.8326 | 25 | 605,651,653,339 | 605 |
| TG (18:1/20:4/22:5) | 972.8008 | 25 | 625,651,673,339 | 625 |
|  | | | | |
| **DG** | **[M+NH_4_] ^+^** |  |  | **NL(FA) -H+NH_4_^+^** |
| DG (20:0/20:0) standard | 698.6649 | 25 | 369,663 | 369 |
| DG (16:0/20:5) | 632.5242 | 25 | 313,359,597 | 313 |
| DG (18:0/18:1) | 640.5876 | 25 | 341,339,605 | 341 |
| DG (16:0/18:1) | 612.5558 | 25 | 313,339,577 | 313 |
| DG (16:0/18:2) | 610.5404 | 25 | 313,337,575 | 313 |
| DG (18:1/18:2) | 636.5558 | 25 | 339,337,601 | 339 |
| DG (20:4/20:4) | 682.5401 | 25 | 361,287 | 361 |
| DG (18:0/22:6) | 686.5719 | 25 | 341,385,651 | 341 |
| DG (18:0/22:5) | 688.5867 | 25 | 341,387,653 | 341 |
| DG (18:1/20:5) | 658.5399 | 25 | 339,359 | 339 |
| DG (18:1/18:1) | 638.5717 | 25 | 339,603,265 | 339 |

Table S1 continued.

| **Lipid Species** | ***m/z* of the adduct** | **Collision energy** | **Ion Fragments used for confirmation** | **Product ion used for quantification** |
| --- | --- | --- | --- | --- |
| **DG** | **[M+NH_4_] ^+^** |  |  | **NL(FA) -H+NH_4_^+^** |
| DG (18:1/20:4) | 660.556 | 25 | 339,361,625 | 339 |
| DG (16:0/20:4) | 634.5407 | 25 | 313,361 | 313 |
|  | | | | |
| **PE** | **[M-H]^-^** |  |  | **Glycerol phosphoethanolamine** |
| PE (14:0/14:0) standard | 634.4455 | 30 | 227,140,424,196 | 196 |
| PE (16:1/18:1) | 714.5079 | 30 | 253,281,140,196 | 196 |
| PE (16:1/16:1) | 686.4769 | 30 | 253,140,196 | 196 |
| PE (16:0/16:1) | 688.4928 | 30 | 253,255,140,196 | 196 |
| PE (17:1/16:0) | 702.5085 | 30 | 267,255,140,196 | 196 |
| PE (16:0/18:2) | 714.5087 | 30 | 279,255,140,196 | 196 |
| PE (17:1/16:1) | 700.4925 | 30 | 267,253,140,196 | 196 |
|  | | | | |
| **PC** | **[M+H]^+^** |  | **[M+HCOO]^-^ fragments** | **phosphocholine head [C_5_H_13_NPO_4_]^+^** |
| PC (20:0/20:0) standard | 846.69 | 30 | 311,536,168 | 184 |
| PC (16:1/20:4) | 780.5529 | 30 | 253,303,168 | 184 |
| PC (22:5/20:4) | 856.5836 | 30 | 303,329,168 | 184 |
| PC (16:1/14:0) | 704.5221 | 30 | 227,253,168 | 184 |
| PC (18:0/16:0) | 762.6005 | 30 | 255,283,168 | 184 |
| PC (16:1/18:2) | 756.5532 | 30 | 253,279,168 | 184 |
|  |  |  |  |  |
| **LPC** | **[M+H]^+^** |  | **[M+HCOO]^-^ fragments** | **phosphocholine head [C_5_H_13_NPO_4_]^+^** |
| LPC 17:0 standard | 510.3554 | 30 | 269,494 | 184 |
| LPC 16:0 | 496.3399 | 30 | 255,480 | 194 |
| LPC 18:0 | 524.3716 | 30 | 283,508 | 184 |

**Table S2: Increased lipid molecular species identified in Figure 2E Venn diagram.** Note: “d” and “e” designations in supplemental table indicate dihydro and alkyl ether, respectively.

| **Only in CFT073 2h** | **Only in CFT073 1h** | **Only in JM109 2h** | **Common in CFT073 2h, CFT073 1h, JM109 2h and JM109 1h** | **Common in CFT073 2h and CFT073 1h** | **Common in CFT073 2h and JM109 2h** | **Common in CFT073 1h, JM109 2h and JM109 1h** | **Common in CFT073 2h, CFT073 1h and JM109 2h** |
| --- | --- | --- | --- | --- | --- | --- | --- |
| 55 | 2 | 2 | 4 | 6 | 1 | 1 | 1 |
| Cer(d16:1/24:1) | PE(16:1e/22:5) | PE(16:0/14:1) | PG(16:0/16:1) | DG(16:0/16:0) | PE(17:1/16:0) | PG(17:1/16:0) | PS(18:0/16:1) |
| Cer(d17:1/16:0) | PE(18:2e/22:6) | PE(17:1/16:1) | PG(16:0/18:1) | DG(16:0/18:1) |  |  |  |
| Cer(d17:1/22:0) |  |  | PG(16:1/18:1) | DG(18:1/18:1) |  |  |  |
| Cer(d18:1/14:0) |  |  | PG(18:1/18:1) | LPC(16:0) |  |  |  |
| Cer(d18:1/16:0) |  |  |  | LPC(18:0) |  |  |  |
| Cer(d18:1/18:0) |  |  |  | PE(16:1/18:1) |  |  |  |
| Cer(d18:1/20:0) |  |  |  |  |  |  |  |
| Cer(d18:1/24:1) |  |  |  |  |  |  |  |
| Cer(d18:1/24:2) |  |  |  |  |  |  |  |
| Cer(d18:2/16:0) |  |  |  |  |  |  |  |
| Cer(d18:2/22:0) |  |  |  |  |  |  |  |
| Cer(d18:2/24:0) |  |  |  |  |  |  |  |
| Cer(d18:2/24:1) |  |  |  |  |  |  |  |
| Cer(d18:2/26:0) |  |  |  |  |  |  |  |
| Cer(d18:2/26:1) |  |  |  |  |  |  |  |
| DG(16:0/16:1) |  |  |  |  |  |  |  |
| DG(16:0/20:4) |  |  |  |  |  |  |  |
| DG(16:0/20:5) |  |  |  |  |  |  |  |
| DG(18:0/16:0) |  |  |  |  |  |  |  |
| DG(18:0/18:0) |  |  |  |  |  |  |  |
| DG(18:0/18:1) |  |  |  |  |  |  |  |
| DG(18:0/18:2) |  |  |  |  |  |  |  |
| DG(18:0/22:6) |  |  |  |  |  |  |  |
| DG(18:1/18:2) |  |  |  |  |  |  |  |
| DG(18:1/20:5) |  |  |  |  |  |  |  |
| DG(18:1/22:0) |  |  |  |  |  |  |  |
| DG(18:1/22:5) |  |  |  |  |  |  |  |
| DG(18:1/24:0) |  |  |  |  |  |  |  |
| DG(18:1/24:1) |  |  |  |  |  |  |  |
| DG(20:4/20:4) |  |  |  |  |  |  |  |
| DG(26:0/18:1) |  |  |  |  |  |  |  |
| LPC(20:3) |  |  |  |  |  |  |  |
| PC(15:0/16:0) |  |  |  |  |  |  |  |
| PC(16:1e/16:0) |  |  |  |  |  |  |  |
| PC(16:1e/18:1) |  |  |  |  |  |  |  |
| PC(16:1e/22:6) |  |  |  |  |  |  |  |
| PC(18:0/16:0) |  |  |  |  |  |  |  |
| PC(20:4/20:4) |  |  |  |  |  |  |  |
| PE(16:0/16:0) |  |  |  |  |  |  |  |
| PE(16:0/16:1) |  |  |  |  |  |  |  |
| PE(16:0/18:1) |  |  |  |  |  |  |  |
| PE(16:0/18:2) |  |  |  |  |  |  |  |
| PE(16:1/14:0) |  |  |  |  |  |  |  |
| PE(16:1/16:1) |  |  |  |  |  |  |  |
| PE(18:0/16:0) |  |  |  |  |  |  |  |
| PE(18:0/16:1) |  |  |  |  |  |  |  |
| PE(18:0/18:2) |  |  |  |  |  |  |  |
| PE(18:1e/16:0) |  |  |  |  |  |  |  |
| PE(20:1e/16:0) |  |  |  |  |  |  |  |
| TG(16:0/16:0/20:4) |  |  |  |  |  |  |  |
| TG(16:0/18:1/18:2) |  |  |  |  |  |  |  |
| TG(16:0/18:1/20:4) |  |  |  |  |  |  |  |
| TG(18:0/18:1/20:4) |  |  |  |  |  |  |  |
| TG(18:0/18:1/22:5) |  |  |  |  |  |  |  |
| TG(18:1/18:1/20:4) |  |  |  |  |  |  |  |

**Table S3: Decreased lipid molecular species identified in Figure 2F Venn diagram.** Note: “e” and “p” designations in supplemental table indicate alkyl ether and plasmalogen, respectively.

| **Only in CFT073 2h** | **Only in CFT073 1h** | **Only in JM109 2h** | **Only in JM109 1h** | **Common in CFT073 1h and JM109 1h** | **Common in CFT073 1h, JM109 1h and JM109 2h** | **Common in CFT073 2h, JM109 1h and JM109 2h** | **Common in JM109 1h and JM109 2h** | **Common in CFT073 1h and JM109 2h** |
| --- | --- | --- | --- | --- | --- | --- | --- | --- |
| 2 | 2 | 1 | 2 | 1 | 1 | 1 | 1 | 1 |
| PE(18:1/20:5) | PE(16:0p/20:4) | DG(18:0/20:5) | DG(16:0/22:4) | DG(18:1/20:4) | DG(17:0/20:4) | DG(18:0/20:4) | DG(18:0/22:5) | PC(18:0/22:6) |
| PG(16:0/14:0) | PE(16:1e/20:4) |  | PC(18:1e/20:4) |  |  |  |  |  |

**Table S4:** **Increased lipid molecular species identified in Figure 5C Venn diagram.** Note: “e” designation in supplemental table indicates alkyl ether. * indicates untargeted analysis did not distinguish *sn*-1 and *sn*-2 aliphatic constituents.

| **Only in CFT073** | **Only in JM109** | **Common in CFT073 and JM109** |
| --- | --- | --- |
| 65 | 10 | 15 |
| DG(16:0/18:1) | DG(18:0/20:5) | DG(16:0/20:4) |
| DG(16:0/18:2) | DG(18:0/22:4) | DG(18:0/22:3) |
| DG(16:0/20:3) | DG(26:0/20:4) | DG(18:0/22:5) |
| DG(16:0/20:5) | PC(38:2e)* | DG(18:0/22:6) |
| DG(16:0/22:4) | PC(42:1)* | DG(18:1/18:1) |
| DG(18:0/18:1) | PE(26:0/18:1) | DG(18:1/20:4) |
| DG(18:0/20:1) | PG(15:0/16:0) | DG(18:1/20:5) |
| DG(18:1/18:2) | PG(16:0/14:0) | DG(20:4/20:4) |
| DG(18:1/22:4) | PG(16:1/14:0) | DG(26:1/16:0) |
| DG(18:1/24:0) | TG(16:1/14:0/14:0) | PG(16:0/16:1) |
| DG(18:2/20:4) |  | PG(16:0/18:1) |
| DG(20:2/18:2) |  | PG(16:1/16:1) |
| DG(20:5/20:4) |  | PG(16:1/18:1) |
| DG(26:0/16:0) |  | PG(17:1/16:0) |
| DG(26:0/18:0) |  | PG(18:1/18:1) |
| DG(26:0/18:2) |  |  |
| DG(26:1/18:1) |  |  |
| DG(28:0/18:1) |  |  |
| PC(17:0/18:1) |  |  |
| PC(30:1)* |  |  |
| PC(33:0)* |  |  |
| PC(34:0)* |  |  |
| PC(34:3)* |  |  |
| PC(36:0)* |  |  |
| PC(36:5)* |  |  |
| PC(37:5)* |  |  |
| PC(38:6e)* |  |  |
| PC(38:7e)* |  |  |
| PC(42:9)* |  |  |
| PE(16:0/16:1) |  |  |
| PE(16:0/18:1) |  |  |
| PE(16:0/18:2) |  |  |
| PE(16:1/16:1) |  |  |
| PE(16:1/18:1) |  |  |
| PE(17:1/16:0) |  |  |
| PE(17:1/16:1) |  |  |
| PG(16:0/16:0) |  |  |
| PG(16:0/16:0) |  |  |
| PG(16:0/16:1) |  |  |
| PG(16:0/18:1) |  |  |
| PG(16:0/18:2) |  |  |
| PG(16:0/18:2) |  |  |
| PG(16:1/16:1) |  |  |
| PG(16:1/18:1) |  |  |
| PG(17:1/16:0) |  |  |
| PG(18:1/18:1) |  |  |
| TG(16:0/16:0/20:4) |  |  |
| TG(16:0/16:1/20:4) |  |  |
| TG(16:0/22:4/22:6) |  |  |
| TG(16:0/22:5/22:6) |  |  |
| TG(17:0/18:1/22:5) |  |  |
| TG(18:0/18:1/20:4) |  |  |
| TG(18:0/18:1/22:5) |  |  |
| TG(18:0/20:4/22:6) |  |  |
| TG(18:1/17:1/18:1) |  |  |
| TG(18:1/18:1/20:4) |  |  |
| TG(18:1/18:2/18:2) |  |  |
| TG(18:1/18:2/20:4) |  |  |
| TG(18:1/18:2/22:6) |  |  |
| TG(18:1/20:3/22:4) |  |  |
| TG(18:1/20:4/20:4) |  |  |
| TG(18:1/20:4/22:5) |  |  |
| TG(18:2/22:6/22:6) |  |  |
| TG(22:5/18:2/22:6) |  |  |
| TG(26:0/16:0/18:1) |  |  |

**Table S5:** **Decreased lipid molecular species identified in Figure 5D Venn diagram.** Note: “d” and “e” designations in supplemental table indicate dihydro and alkyl ether, respectively. * indicates untargeted analysis did not distinguish *sn*-1 and *sn*-2 aliphatic constituents.

| **Only in CFT073** | **Only in JM109** |
| --- | --- |
| 10 | 22 |
| DG(18:0/20:4) | PC(18:0/22:5) |
| PE(16:0e/16:0) | Cer(d17:1/16:0) |
| PE(16:0e/18:1) | Cer(d18:0/16:0) |
| PE(18:0e/18:2) | Cer(d18:2/16:0) |
| PS(18:0/18:2) | Cer(d18:2/18:0) |
| PS(18:0/20:4) | LPC(20:4) |
| PS(18:1/18:1) | PC(16:1e/16:1) |
| TG(12:0/12:0/12:0) | PC(16:1e/18:1) |
| TG(16:0/12:0/12:0) | PC(36:3e)* |
| TG(16:0/14:0/16:1) | PC(36:4e)* |
|  | PC(38:1e)* |
|  | PC(38:4)* |
|  | PC(38:6e)* |
|  | PE(16:1e/20:5) |
|  | PE(20:1e/20:2) |
|  | TG(16:0/18:2/18:2) |
|  | TG(18:0/16:0/20:3) |
|  | TG(18:0/18:1/18:2) |
|  | TG(18:1/18:2/18:2) |
|  | TG(18:2/18:2/18:2) |
|  | TG(18:3/18:2/18:2) |
|  | TG(8:0/10:0/10:0) |

**Table S6:** **Increased lipid molecular species identified in Figure 7A Venn diagram.** Note: “d” and “e” designations in supplemental table indicate dihydro and alkyl ether, respectively. * indicates untargeted analysis did not distinguish *sn*-1 and *sn*-2 aliphatic constituents.

| **Only in CFT073+Neu+EA 2h** | **Only in CFT073+EA 1h** | **Only in CFT073+EA 2h** | **Common in CFT073+Neu+EA and CFT073+EA 2h** | **Common in CFT073+ EA 1h and CFT073+EA 1 h** | **Common in CFT073+Neu+EA 2h and CFT073+EA 1h** | **Common in CFT073+Neu+EA, CFT073+EA 1h and CFT073 2h** |
| --- | --- | --- | --- | --- | --- | --- |
| 55 | 2 | 39 | 17 | 4 | 1 | 7 |
| DG(16:0/18:2) | PE(16:1e/22:5) | Cer(d16:1/24:1) | DG(16:0/20:4) | DG(16:0/16:0) | PG(17:1/16:0) | DG(16:0/18:1) |
| DG(16:0/20:3) | PE(18:2e/22:6) | Cer(d17:1/16:0) | DG(16:0/20:5) | LPC(16:0) |  | DG(18:1/18:1) |
| DG(16:0/22:4) |  | Cer(d17:1/22:0) | DG(18:0/18:1) | LPC(18:0) |  | PE(16:1/18:1) |
| DG(18:0/20:1) |  | Cer(d18:1/14:0) | DG(18:0/22:6) | PS(18:0/16:1) |  | PG(16:0/16:1) |
| DG(18:0/22:3) |  | Cer(d18:1/16:0) | DG(18:1/18:2) |  |  | PG(16:0/18:1) |
| DG(18:0/22:5) |  | Cer(d18:1/18:0) | DG(18:1/20:5) |  |  | PG(16:1/18:1) |
| DG(18:1/20:4) |  | Cer(d18:1/20:0) | DG(18:1/24:0) |  |  | PG(18:1/18:1) |
| DG(18:1/22:4) |  | Cer(d18:1/24:1) | DG(20:4/20:4) |  |  |  |
| DG(18:2/20:4) |  | Cer(d18:1/24:2) | PE(16:0/16:1) |  |  |  |
| DG(20:2/18:2) |  | Cer(d18:2/16:0) | PE(16:0/18:1) |  |  |  |
| DG(20:5/20:4) |  | Cer(d18:2/22:0) | PE(16:0/18:2) |  |  |  |
| DG(26:0/16:0) |  | Cer(d18:2/24:0) | PE(16:1/16:1) |  |  |  |
| DG(26:0/18:0) |  | Cer(d18:2/24:1) | PE(17:1/16:0) |  |  |  |
| DG(26:0/18:2) |  | Cer(d18:2/26:0) | TG(16:0/16:0/20:4) |  |  |  |
| DG(26:1/16:0) |  | Cer(d18:2/26:1) | TG(18:0/18:1/20:4) |  |  |  |
| DG(26:1/18:1) |  | DG(16:0/16:1) | TG(18:0/18:1/22:5) |  |  |  |
| DG(28:0/18:1) |  | DG(18:0/16:0) | TG(18:1/18:1/20:4) |  |  |  |
| PC(17:0/18:1) |  | DG(18:0/18:0) |  |  |  |  |
| PC(30:1)* |  | DG(18:0/18:2) |  |  |  |  |
| PC(33:0)* |  | DG(18:1/22:0) |  |  |  |  |
| PC(34:0)* |  | DG(18:1/22:5) |  |  |  |  |
| PC(34:3)* |  | DG(18:1/24:1) |  |  |  |  |
| PC(36:0)* |  | DG(26:0/18:1) |  |  |  |  |
| PC(36:5)* |  | LPC(20:3) |  |  |  |  |
| PC(37:5)* |  | PC(15:0/16:0) |  |  |  |  |
| PC(38:6e)* |  | PC(16:1e/16:0) |  |  |  |  |
| PC(38:7e)* |  | PC(16:1e/18:1) |  |  |  |  |
| PC(42:9)* |  | PC(16:1e/22:6) |  |  |  |  |
| PE(17:1/16:1) |  | PC(18:0/16:0) |  |  |  |  |
| PG(16:0/16:0) |  | PC(20:4/20:4) |  |  |  |  |
| PG(16:0/16:0) |  | PE(16:0/16:0) |  |  |  |  |
| PG(16:0/16:1) |  | PE(16:1/14:0) |  |  |  |  |
| PG(16:0/18:1) |  | PE(18:0/16:0) |  |  |  |  |
| PG(16:0/18:2) |  | PE(18:0/16:1) |  |  |  |  |
| PG(16:0/18:2) |  | PE(18:0/18:2) |  |  |  |  |
| PG(16:1/16:1) |  | PE(18:1e/16:0) |  |  |  |  |
| PG(16:1/16:1) |  | PE(20:1e/16:0) |  |  |  |  |
| PG(16:1/18:1) |  | TG(16:0/18:1/18:2) |  |  |  |  |
| PG(17:1/16:0) |  | TG(16:0/18:1/20:4) |  |  |  |  |
| PG(18:1/18:1) |  |  |  |  |  |  |
| TG(16:0/16:1/20:4) |  |  |  |  |  |  |
| TG(16:0/22:4/22:6) |  |  |  |  |  |  |
| TG(16:0/22:5/22:6) |  |  |  |  |  |  |
| TG(17:0/18:1/22:5) |  |  |  |  |  |  |
| TG(18:0/20:4/22:6) |  |  |  |  |  |  |
| TG(18:1/17:1/18:1) |  |  |  |  |  |  |
| TG(18:1/18:2/18:2) |  |  |  |  |  |  |
| TG(18:1/18:2/20:4) |  |  |  |  |  |  |
| TG(18:1/18:2/22:6) |  |  |  |  |  |  |
| TG(18:1/20:3/22:4) |  |  |  |  |  |  |
| TG(18:1/20:4/20:4) |  |  |  |  |  |  |
| TG(18:1/20:4/22:5) |  |  |  |  |  |  |
| TG(18:2/22:6/22:6) |  |  |  |  |  |  |
| TG(22:5/18:2/22:6) |  |  |  |  |  |  |
| TG(26:0/16:0/18:1) |  |  |  |  |  |  |

**Table S7:** **Decreased lipid molecular species identified in Figure 7B Venn diagram.** Note: “e” and “p” designations in supplemental table indicate alkyl ether and plasmalogen, respectively.

| **Only in CFT073+Neu+EA 2h** | **Only in CFT073+EA 1h** | **Only in CFT073+EA 2h** | **Common in CFT073+Neu+EA 2h and CFT073+EA 2h** | **Common in CFT073+EA 1h and CFT073+EA 2h** |
| --- | --- | --- | --- | --- |
| 9 | 3 | 2 | 1 | 2 |
| PE(16:0e/16:0) | DG(18:1/20:4) | DG(18:0/20:5) | DG(18:0/20:4) | DG(17:0/20:4) |
| PE(16:0e/18:1) | PE(16:0p/20:4) | DG(18:0/22:5) |  | PC(18:0/22:6) |
| PE(18:0e/18:2) | PE(16:1e/20:4) |  |  |  |
| PS(18:0/18:2) |  |  |  |  |
| PS(18:0/20:4) |  |  |  |  |
| PS(18:1/18:1) |  |  |  |  |
| TG(12:0/12:0/12:0) |  |  |  |  |
| TG(16:0/12:0/12:0) |  |  |  |  |
| TG(16:0/14:0/16:1) |  |  |  |  |

**Table S8:** **Increased lipid molecular species identified in Figure 7C Venn diagram.** Note: “e” designation in supplemental tables indicates alkyl ether. * indicates untargeted analysis did not distinguish *sn*-1 and *sn*-2 aliphatic constituents.

| **Only in JM109+Neu+EA 2h** | **Only in JM109+EA 2h** | **Common in JM109+Neu+EA 2h, JM109+EA 1h and JM109+EA 2h** |
| --- | --- | --- |
| 20 | 4 | 5 |
| DG(16:0/20:4) | PE(16:0/14:1) | PG(16:0/16:1) |
| DG(18:0/20:5) | PE(17:1/16:0) | PG(16:0/18:1) |
| DG(18:0/22:3) | PE(17:1/16:1) | PG(16:1/18:1) |
| DG(18:0/22:4) | PS(18:0/16:1) | PG(17:1/16:0) |
| DG(18:0/22:5) |  | PG(18:1/18:1) |
| DG(18:0/22:6) |  |  |
| DG(18:1/18:1) |  |  |
| DG(18:1/20:4) |  |  |
| DG(18:1/20:5) |  |  |
| DG(20:4/20:4) |  |  |
| DG(26:0/20:4) |  |  |
| DG(26:1/16:0) |  |  |
| PC(38:2e)* |  |  |
| PC(42:1)* |  |  |
| PE(26:0/18:1) |  |  |
| PG(15:0/16:0) |  |  |
| PG(16:0/14:0) |  |  |
| PG(16:1/14:0) |  |  |
| PG(16:1/16:1) |  |  |
| TG(16:1/14:0/14:0) |  |  |

**Table S9:** **Decreased lipid molecular species identified in Figure 7D Venn diagram.** Note “d” and “e” designations in supplemental table indicate dihydro and alkyl ether, respectively. * indicates untargeted analysis did not distinguish *sn*-1 and *sn*-2 aliphatic constituents.

| **Only in JM109+Neu+EA 2h** | **Only in JM109+EA 1h** | **Only in JM109+EA 2h** | **Common in JM109+EA 1h and JM109+EA 2h** |
| --- | --- | --- | --- |
| 22 | 3 | 2 | 3 |
| PC(18:0/22:5) | DG(16:0/22:4) | DG(18:0/20:5) | DG(17:0/20:4) |
| Cer(d17:1/16:0) | DG(18:1/20:4) | PC(18:0/22:6) | DG(17:0/20:4) |
| Cer(d18:0/16:0) | PC(18:1e/20:4) |  | DG(18:0/20:4) |
| Cer(d18:2/16:0) |  |  | DG(18:0/22:5) |
| Cer(d18:2/18:0) |  |  |  |
| LPC(20:4) |  |  |  |
| PC(16:1e/16:1) |  |  |  |
| PC(16:1e/18:1) |  |  |  |
| PC(36:3e)* |  |  |  |
| PC(36:4e)* |  |  |  |
| PC(38:1e)* |  |  |  |
| PC(38:4)* |  |  |  |
| PC(38:6e)* |  |  |  |
| PE(16:1e/20:5) |  |  |  |
| PE(20:1e/20:2) |  |  |  |
| TG(16:0/18:2/18:2) |  |  |  |
| TG(18:0/16:0/20:3) |  |  |  |
| TG(18:0/18:1/18:2) |  |  |  |
| TG(18:1/18:2/18:2) |  |  |  |
| TG(18:2/18:2/18:2) |  |  |  |
| TG(18:3/18:2/18:2) |  |  |  |
| TG(8:0/10:0/10:0) |  |  |  |
